# Gamification of e-learning in higher education: a systematic literature review

**DOI:** 10.1186/s40561-023-00227-z

**Published:** 2023-01-31

**Authors:** Amina Khaldi, Rokia Bouzidi, Fahima Nader

**Affiliations:** Ecole Nationale Supérieure d’Informatique ESI, Ex INI, Algiers, Algeria

**Keywords:** Gamification, Higher education, Tertiary education, e-learning, Digital learning environments, Systematic review

## Abstract

In recent years, university teaching methods have evolved and almost all higher education institutions use e-learning platforms to deliver courses and learning activities. However, these digital learning environments present significant dropout and low completion rates. This is primarily due to the lack of student motivation and engagement. Gamification which can be defined as the application of game design elements in non-game activities has been used to address the issue of learner distraction and stimulate students’ involvement in the course. However, choosing the right combination of game elements remains a challenge for gamification designers and practitioners due to the lack of proven design approaches, and there is no one-size-fits-all approach that works regardless of the gamification context. Therefore, our study focused on providing a comprehensive overview of the current state of gamification in online learning in higher education that can serve as a resource for gamification practitioners when designing gamified systems. In this paper, we aimed to systematically explore the different game elements and gamification theory that have been used in empirical studies; establish different ways in which these game elements have been combined and provide a review of the state-of-the-art of approaches proposed in the literature for gamifying e-learning systems in higher education. A systematic search of databases was conducted to select articles related to gamification in digital higher education for this review, namely, Scopus and Google Scholar databases. We included studies that consider the definition of gamification as the application of game design elements in non-game activities, designed for online higher education. We excluded papers that use the term of gamification to refer to game-based learning, serious games, games, video games, and those that consider face-to-face learning environments. We found that PBL elements (points, badges, and leaderboards), levels, and feedback and are the most commonly used elements for gamifying e-learning systems in higher education. We also observed the increasing use of deeper elements like challenges and storytelling. Furthermore, we noticed that of 39 primary studies, only nine studies were underpinned by motivational theories, and only two other studies used theoretical gamification frameworks proposed in the literature to build their e-learning systems. Finally, our classification of gamification approaches reveals the trend towards customization and personalization in gamification and highlights the lack of studies on content gamification compared to structural gamification.

## Introduction

In recent years, most universities use e-learning platforms to deliver courses. Teaching in the form of e-learning is a modern supplement, and sometimes even an alternative to traditional education (Górska, 2016). Especially since the last few years, with the spread of the Covid-19 crisis, higher education institutions had to shift from traditional teaching to online teaching as an alternative to resume learners' learning (Sofiadin & Azuddin, 2021). However, over time, these digital environments brought several challenges. On one hand, student motivation decreases, resulting in a lack of engagement and participation in courses. On the other hand, instructors struggle to maintain learners’ attention, leading to the eventual abandonment of online education systems. To solve this problem and create engaging e-learning platforms, the gamification technique was proposed.

Game technologies create opportunities for higher education institutions to redesign and innovate their e-learning models to support learning experiences among learners (Alhammad & Moreno, 2018). The introduction and growing expansion of gamification in education and learning contexts promotes critical reflection on the development of projects that transform students’ learning experiences (Garone & Nesteriuk, 2019). However, is it that simple to create effective gamified e-learning systems especially in the context of higher education?

Early applied work on gamification of educational settings suggested positive-learning, but mixed results have been obtained (Seaborn & Fels, 2015). While gamification in general learning systems is known to have a positive impact on student motivation, evidence on its effectiveness in higher education settings is mixed and still uncertain due to the complicated environment in the higher education context. First, the level of difficulty of study is higher at the university than at lower levels of education, and students are more aware of the importance of education they have chosen (Urh et al., 2015). Moreover, tertiary education is characterized by the variety of students’ profiles, needs and learning methods; thereby, each game element and even each combination of game elements affects each student differently. Given this diversity of features in the higher education context and the increasing number of inter- and multidisciplinary programs, the process of applying gamification is becoming more complex.

The purpose of this systematic review was to provide a comprehensive overview of the current state of gamification in e-learning in higher education. We focused on identifying how designers currently deal with gamification in the digital higher education context, what game elements they use, how these elements are combined, and what gamification theories are used. In addition, this study sought to find data on existing gamification approaches in the literature, especially those suggested to be applied in digital higher education. Our study differs from previous studies in several ways. In our study, we first wanted to compare our results with previous research’s results that addressed the same research questions in terms of trends in the use of game elements, i.e. whether designers who develop gamified e-learning systems still use classic game elements such as points, badges, and leaderboards, or whether they expand the list of game elements used to include deeper game elements like challenges, storytelling, and so on. We then focused on the underpinning gamification theories used in empirical work, and specifically we sought to understand whether empirical research is beginning to use the various gamification frameworks available in the literature, or whether it is still relying on theories and methods that are highly theoretical and do not provide clear guidance to designers when choosing the right set of game elements (Toda et al., 2020). Also, in our study, we sought to find out how game elements are combined in gamified learning systems in higher education. Previous studies have not fully explored this point, with the exception of the study (Dichev & Dicheva, 2017). Finally, we proposed a classification of gamification approaches proposed in the context of e-learning in higher education based on several relevant criteria.

The remainder of this manuscript has the following structure. "Related works" section, briefly reviews some of the most relevant review papers. "Systematic literature review methodology" section, systematic literature review methodology, presents the approach we followed in conducting our paper retrieval. "Results of the search" section, results of the research, presents responses to our defined research questions. "Discussion and limitations" section is dedicated for discussion of the results; and finally, we conclude.

## Related works

### Prior reviews

This section briefly reviews some of the relevant literature reviews on gamification in higher education related to the topic of our systematic review. The objective is to be able to compare our findings later in the results section to prior reviews’ findings and to shed a more realistic light on any advances in gamification in e-learning in the context of higher education.

Dichev and Dicheva (2017) critically reviewed the advancement of educational gamification. This review paper was the only one to address the issue of combining game elements in gamified learning systems. The authors found that in all reviewed works, no justification is given for the selection of particular game elements. The study concluded that there is a need for further studies to improve our understanding of how individual game elements are associated with behavioral and motivational outcomes and how they function in an educational context.

Ozdamli (2018) examined 313 studies on gamification in education. It used content analysis to determine trends in gamification research. The study sought to determine the distribution of empirical research based on a variety of criteria, namely: distribution of studies based on years, number of authors, type of publication, paradigms, research sample, environments, theory/model/strategy, learning area and distribution of game components, mechanics and dynamics. The author found that motivational theories are the most frequently used approach in gamification studies and that the most frequently used game components are goals, rewards and progression sticks.

Khalil et al. (2018) reviewed the state of the art on gamification in MOOCs (Massive Open Online Course) by answering eight research questions. One of these questions sought to identify elements of gamification that have been implemented or proposed for implementation in MOOCs. The study found that the most commonly used elements in the application of gamification in MOOCs are badges, leaderboards, progress, and challenges. According to the study, progress and challenges are used more frequently in MOOCs than points.

The paper (Alhammad & Moreno, 2018) studied gamification in the context of software engineering (SE) education. The study sought to understand how gamification was applied in the SE curriculum and what game elements were used. The study identified four gamification approaches from the primary studies analyzed: papers that implemented gamification by following an existing gamification approach in the literature, papers that adapted psychological and educational theories as gamification approaches, papers that designed and followed their own gamification approach, and finally, papers that did not follow any specific gamification approach. In addition, leaderboards, points and levels were found to be the most frequently used gaming components. Similarly, challenges, feedback, and rewards were the most commonly used mechanics, and progression was the most commonly used dynamic.

Majuri et al. (2018) reviewed 128 empirical research papers in the literature on gamification in education and learning. It was found that points, challenges, badges and leaderboards are the most commonly used gamification affordances in education which are affordances that refer to achievement and progression while social and immersion-oriented affordances are much less common.

In the paper (Zainuddin et al., 2020), the authors addressed a research question related to our research area, namely the underlying theoretical models used in gamification research. It was found that in the studies that implicitly mention their theoretical underpinnings, self-determination theory is the most commonly used, followed by flow theory and goal-setting, while the other studies do not provide any theoretical content.

More recently, van Gaalen et al. (2021) reviewed 44 research studies in the health professions education literature. The study addressed the question of what game attributes are used in gamified environments, and sought to understand the use of theory throughout the gamification process. The study used Landers (2014)’s framework to categorize the identified game elements into game attributes and revealed that in most reviewed studies the game attributes ‘assessment’ and/or ‘conflict/challenge’ were embedded in the learning environment. Regarding the use of theory in gamification processes, most of the identified studies on gamification in health professions education were not theory-based, or theoretical considerations were not included or not yet developed.

Finally, the authors of the paper (Kalogiannakis et al., 2021) performed a systematic literature review on gamification in science education by reviewing 24 empirical research papers. A research question related to our field of study was addressed in this review, namely, what learning theory is used, and what game elements are incorporated into gaming apps. The findings of the studyshowed that most articles did not provide details about the theoretical content or the theory on which they were based. The few articles that used theoretical frameworks were based on self-determination theory SDT, flow theory, goal-setting theory, cognitive theory of multimedia learning and motivation theory. In addition, the study found that the most common game elements and mechanics used in gamified science education environments were competitive setup, leaderboards, points and levels.

## Systematic literature review methodology

In this paper of systematic review, we followed a methodology to identify how gamification technique has been used in digital learning environments, specifically in higher education. We sought to identify the game elements that have been used the most, the way they have been combined, and the different frameworks proposed in the literature for gamification of e-learning systems in higher education. A systematic literature review is a means of identifying, evaluating and interpreting all available research relevant to a particular research question, or topic area, or phenomenon of interest (Kitchenham, 2004). Kitchenham (2004) summarizes the stages of a systematic review in three main phases: Planning the Review, Conducting the Review, and Reporting the Review. The first phase ‘Planning the Review’ includes the formulation of research questions, identification of key concepts and constructing the search queries. The second phase ‘Conducting the Review’ consists on study selection based on inclusion and exclusion criteria. Finally, the third phase ‘Reporting the Review’ relates to data extraction and responding to research questions. In the following, we detail the main steps of each phase.

### Search strategy

We started by identifying the main goal of this systematic literature review by clearly formulating the following research questions:

1. Which game elements and gamification theories are used in gamified learning systems?
2. How these game elements are combined?
3. Which gamification design approaches are available in the literature?

Then, we constructed a list of key concepts that are: gamification, e-learning and higher education. After that, we identified the alternative terms for each of the key concepts as some authors may refer to the same concept using a different term. For the concept of gamification, we identified this list of free text terms: gamify, game elements, game dynamics, game mechanics, game components, game aesthetics and gameful. For the two other concepts of e-learning and higher education, we identified these terms: education, educational, learning, teaching, course, syllabus, syllabi, curriculum, and curricula.

We formulated two search queries based on the terms identified previously:

1. For research questions 1and 2:

(gamif* OR gameful OR “game elements” OR “game mechanics” OR “game dynamics” OR “game components” OR “game aesthetics”) AND (education OR educational OR learning OR teaching OR course OR syllabus OR syllabi OR curriculum OR curricula).

(2) For research question 3:

(gamif* OR gameful OR “game elements” OR “game mechanics” OR “game dynamics” OR “game components” OR “game aesthetics”) AND (education OR educational OR learning OR teaching OR course OR syllabus OR syllabi OR curriculum OR curricula) AND (framework OR method OR design OR model OR approach OR theory OR strategy).

We conducted our research by searching the databases using the search query formulated previously. We performed our search in the Scopus and Google Scholar databases as the first is one of the most professional indexing databases and the second is the most popular, so it helps to identify further eligible studies. The search was performed in December 2021. Although the Scopus database indexed the publication abstracts, most of the articles were not available through Scopus, and the articles were retrieved from the following publishers:

- IEEE,
- Springer,
- ACM,
- JSTOR,
- SEMANTIC SCHOLAR,
- (Hallifax et al. ) SAGE,
- Science Direct.

The exception was some articles that could not be accessed. We also performed a backward snowballing search to identify further relevant studies by scanning and searching the references of papers marked as potentially relevant (Dichev & Dicheva, 2017; Mora et al., 2017; Gari & Radermacher, 2018; Khalil et al., 2018; Ozdamli, 2018; Subhash & Cudney, 2018; da Silva et al., 2019; Hallifax et al., 2019a, 2019b; Legaki & Hamari, 2020; Zainuddin et al., 2020; Saleem et al., 2021; Swacha, 2021; van Gaalen et al., 2021) in search of other relevant studies.

### Inclusion and exclusion criteria

In the following table, we summarized the inclusion and exclusion criteria that we considered when we screened full text articles (Table 1).

**Table 1:** Inclusion and exclusion criteria

Criteria	Inclusion	Exclusion
Subject	Gamification (defined as the using of game elements in a non-game context)	Using gamification to refer to game-based learning, serious games, games, video games
Context	Online learning	Conventional learning
Educational level	Higher education	Other settings different from higher education (e.g., work, medicine, elementary school) or no specification about the educational level
Participants	Undergraduate or graduate students	Professors, managerial levels

### Study selection

To select the relevant studies for this systematic review, a manual screening was performed. First, we reviewed the titles and abstracts of different records that were retrieved. Then, citations were imported to Endnote and duplicate records were removed. After that, we read the full text of all retained articles for inclusion and exclusion based on the eligibility criteria. In case of uncertainty, discussion was organized with the research team to reach consensus about the articles in question.

### Data extraction

We developed a data extraction form that was refined and discussed until consensus was obtained. The extraction form was then used by the review author to extract data from all included studies. In this part of this paper, we have considered two types of papers: papers representing case studies to extract the game elements used in the developed e-learning systems, the underpinning theories behind the gamification process and the way game elements were combined with each other. The second type of retrieved papers is about framework proposals, from which we could identify models, approaches, and design processes proposed in the literature for gamifying digital learning environments in tertiary education level.

## Results of the search

### General results

**Fig. 1:**
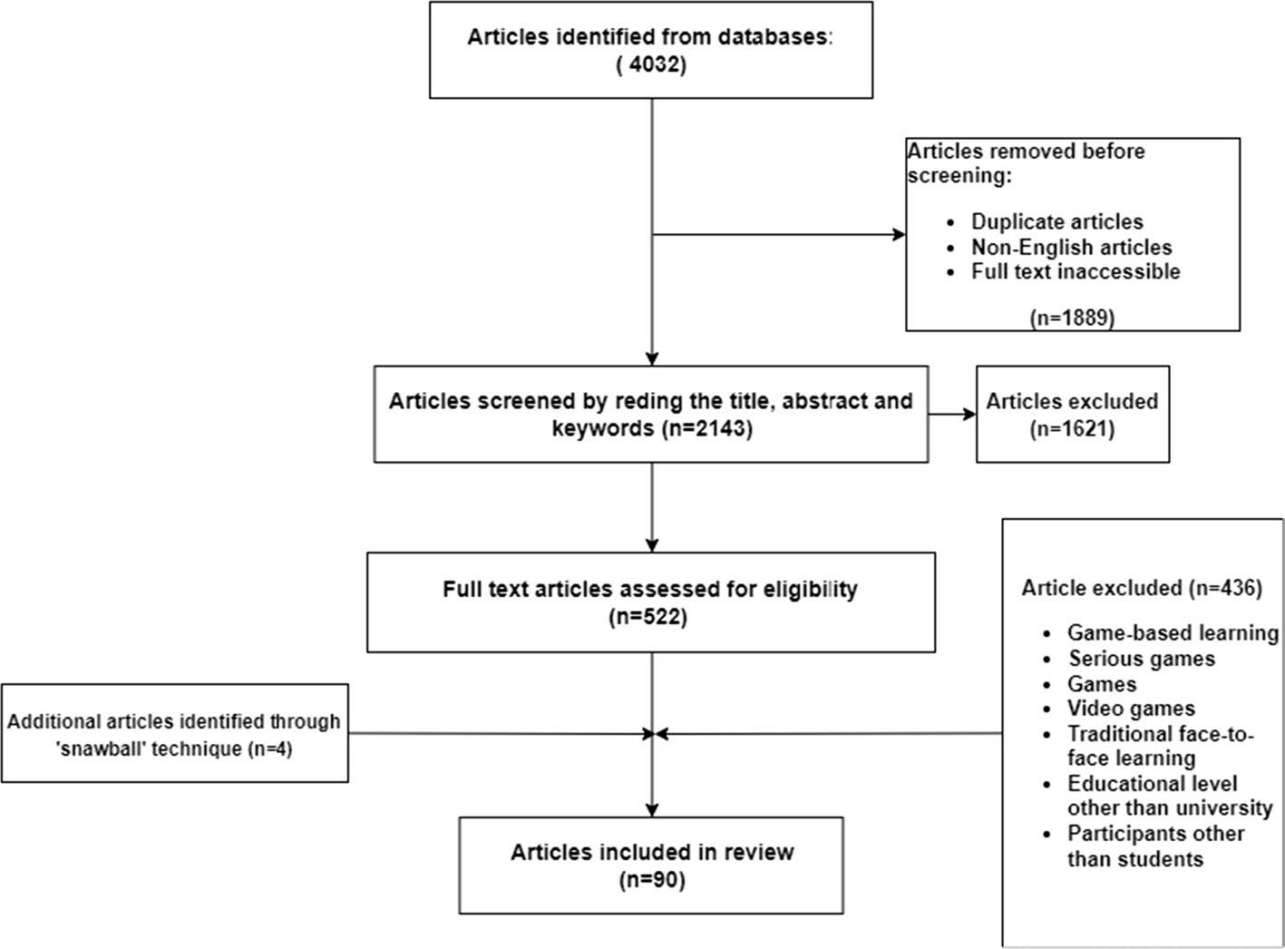
Flow diagram of the articles selection process

In this literature review, we reported the most extensive overview of the empirical research literature on gamification of e-learning in higher education to date. The selection process of relevant studies is shown in Fig. 1. We analyzed a total of 90 papers to respond to the three research questions formulated previously. First, we retrieved 39 papers in the form of empirical studies carried out at university level and analyzed them to identify what game elements are used, what gamification theories are used to guide the gamification process, and how these game elements are combined. We then identified a variety of 51 papers of type theoretical proposals intended to guide the gamification process. Since higher education is part of general learning systems, we included in this review papers that propose gamification approaches for general contexts and general learning systems. Indeed, we identified 16 papers for general application of gamification, 18 papers for gamifying general learning systems and 17 approaches intended to be applied to e-learning systems in higher education.

### Answering research questions

In following, we will answer the three research questions formulated at the beginning of this article:

#### **RQ1**

Which game elements and gamification theories are used in gamified learning systems?

Education applications of gamification refer to using game elements for scholastic development in formal and informal settings (Seaborn & Fels, 2015). In our case, we were interested in collecting relevant experimental studies on gamification of e-learning systems in higher education. In the following table (Table 2), we list and examine 39 experimental studies that have implemented a digital learning system at the higher education level to answer **RQ1**. For each study, we analyzed the game elements that were incorporated and the gamification approaches that were followed during the gamification process. For ease of reference, the game elements that were used in e-learning systems to improve student engagement and the underpinning theories are summarized in Table 2. More detailed descriptions of the 39 empirical studies are presented in “Appendix”.

**Table 2:** Experimental studies on gamification of e-learning in higher education

Paper	Game elements	Underpinning theories
1. Romero-Rodriguez et al. (2019)	Badges**,** Leaderboards, Challenges	A method based on principles established by the paper (Llorens-Largo et al., 2016) was considered when designing the gamified strategies of the 12 MOOCs, namely: Simplicity, feedback, real time, progress, autonomy, individual responsibility. To analyze and evaluate the gamified platform of the energy sustainability-related MOOCs, the study used E-MIGA theoretical taxonomy: Integrated theoretical gamification model in e-learning environments which is proposed by (Dicheva, Dichev et al. 2015)
2. Bernik et al. (2019)	Avatars, Points, Badges, Feedback, Challenges, Simplified graphical interface, Dynamic graphical interface, Story (narrative), Epic meaning, Social networks and web services, Visualization of all obligations, Advancements within the e-course, E-course completion status, Synchronous communication chat, Asynchronous communication forum, Nonlinear use of teaching materials, Collaboration, Interactive repetition and assessment, Top listing and ranking of students, Detection of systems and teaching materials, Elements of surprises within the e-module, Conditional access to teaching materials, Countdown of time	A conceptual model for gamifying e-learning courses was developed based on the work of authors like (Schonfeld, 2010), (Deterding et al., 2011a, 2011b; Deterding, 2012), (Werbach & Hunter, 2012) and (Chou, 2015)
3. Facey-Shaw et al. (2020)	Badges	A gameful design based on self-determination theory SDT (Ryan & Deci, 2000) which provides a framework for examining human motivation through its focus on innate psychological needs (competence, autonomy and relatedness)
4. Bernik et al. (2017)	Avatars, Achievements, Challenges, Behavioral momentum, Productivity, Ownership, Points, Bonuses, Introduction with the information, "Combo" effect × 3, Joint collaboration, Regular rewarding, Status, Epic meaning, Surprise, Advancement, Tasks and challenges, Endless duration of the game, Levels, Loss of aversion, Conscious risk, Optimism, "Addiction"/Commitment to the game	Used gamification mechanics and aesthetics derived from (Nielson, 2017) and (Schonfeld, 2010)
5. Guérard-Poirier et al. (2020)	Checklist (progression), Feedback**,** Points, Scoreboard**,** Progress bar**,** Leaderboard	Not specified
6. Kasinathan et al. (2018)	Scoreboard (points)**,** Time progress bar (time for each question)**,** Challenges	Not specified
7. Kyewski and Krämer (2018)	Badges	Self-determination theory SDT (Ryan & Deci, 2000) Social comparison theory
8. Dikcius et al. (2021)	Rewards	The social exchange theory (SET) The cognitive evaluation theory (CET)
9. Yildirim (2017)	Emotions, Constraints in design, Advancement structure, Narration technique, Chance, Competition, Cooperation, Exchange, Challenge, Points, Badges, Levels, XP, Leaderboards, Medals	A method combining gamification principles for determining additional educational attainments and the framework (Allen, 2007) to balance the difficulty of levels
10. Fajiculay et al. (2017)	Badges, Challenges	Not specified
11. Pilkington (2018)	Points, Badges, Leaderboard**,** Levels**,** avatars**,** Individual and group feedback**,** Journey motif (narrative)	SDT (Self-determination Theory) perspective (Ryan & Deci, 2000) Guided didactic conversation
12. Khaleel et al. (2019)	Stages, Time (countdown), Points, Badges, Leaderboards, Levels	A gamification model was constructed according to student preferences
13. Pérez-López et al. (2017)	Settings, Challenges & missions, Scores, Levels, Rewards, Commitments, Atmosphere, Objectives	Followed the guidelines given by (Kapp 15 mai 2012)
14. Tsay et al. (2018)	Challenges, Freedom to fail, Free to choose, Feedback mechanism, Sense of autonomy, Badges, Content unlocking, Leaderboards, Levels of learning tasks, Competition, Cooperation, Social engagement, Time-based activities	Situated motivational affordance theory (Deterding et al., 2011a, 2011b) User-centered design (Nicholson, 2012)
15. Aşıksoy (2018)	Time limit, XP Points, Badges, Leaderboards, Levels, Feedback	Not specified
16. Khaleel et al. (2020)	Points, Leaderboard, Badges	Not specified
17. Gunawan and Jupiter (2018)	Challenges, Points, Badges, leaderboard	Not specified
18. Bilgin and Gul (2020)	Challenges/tasks, (individual, group, social) Points, (group, individual) leaderboards, Badges, Feedback, Goals, Characters (avatars), Rules, Collaboration/ social activities, Competition, Levels	Used principles from: (Kapp 15 mai, 2012) (Simões et al., 2013)
19. Buckley and Doyle (2017)	Achievements, Avatars, Badges, Boss fights, Collections, Combat content unlocking, Gifting, Leaderboards levels, Points, Levels, Points, Quests social graphs, Teams, virtual goods	Not specified
20. Sanchez et al. (2020)	Feedback (encouraging messages), Progress bar, Wager option	Theory of gamified learning (Landers, 2014)
21. Asiksoy and Canbolat (2021)	Badges, Experience points, Leaderboards, Levels, Instant feedback	Not specified
22. Adams and Du Preez (2021)	Points, Badges, Leaderboards, Levels, Clear goals, Feedback, Rewards, Progress bar, Challenges, Relationships, Cooperation, Competition, Teams	Guidelines offered by: (Kapp 15 mai 2012) (Werbach & Hunter, 2012)
23. Garnett and Button (2018)	Digital badges	Not specified
24. Castro and Gonçalves (2018)	Progress bars, Level up, Ranking, badges	Not specified
25. Coleman (2018)	Badges	Not specified
26. Ropero-Padilla et al. (2021)	Freedom of choice (creating groups), Meaningful purpose (customizing groups: using avatars for each group), Feedback, visibility of progress and path to destination, Ranking score	Not specified
27. Gündüz and Akkoyunlu (2020)	Points, Levels, Badges and achievements, Collections, Weekly and general leaderboards teammates and statistical graphs	Not specified
28. Milenković et al. (2019)	Badges, Leaderboards, Quests, competition	Not specified
29. Donath et al. (2020)	Quests, Challenges, Feedback, progress bar, Badges using BadgR.io system, Level up! Plugin that extends the use of: Experience points Levels Leaderboards Avatars	A conceptual design is proposed to model the learner’s journey using gamification elements, this approach talks about the gamification concepts that are suitable to use for each phase of the learning process so as to meet the education sustainable development needs
30. Pakinee and Puritat (2021)	Points, Levels, Leaderboards, Progress bars, Avatars, Challenges	A framework was adapted based on methods proposed by Alcivar and Abad (2016) and Cechetti, Bellei et al. (2019)
31. van Roy and Zaman (2019)	Challenges, Leaderboards, Badges, Group competition, Group points, Podium like leaderboard, Avatars (group profile with name and logo)	A framework based on Self Determination Theory (Ryan & Deci, 2000) and (van Roy & Zaman, 2017) used as guides to design the gamified platform
32. Ahmed and Asiksoy (2021)	Badges, Experience points, Leaderboards, Levels, Feedback, Timers	Not specified
33. Marín et al. (2019)	Points, Medals, Challenges, Leaderboards, Keys to unlock video lessons	Used MDA framework described in Hunicke et al. (2004)
34. De-Marcos et al. (2020)	Badges, Achievements, Points, leaderboard	A design process based on Self Determination theory SDT (Ryan & Deci, 2000)’s guidelines for the inclusion and design of gamified social elements
35. Donnermann et al. (2021)	Points, Badges	A gamification design based on guidelines from van Roy and Zaman (2017) and Aparicio, Vela et al. (2012)
36. Dias (2017)	Challenges, Points, Badges, Personalized feedback, Leaderboards	Based on Huang and Soman’ gamification process (Wendy Hsin-Yuan Huang 2013) which is a five step process
37. Smith (2017)	Challenge, Feedback	Theory of gamified learning (Landers, 2014) was considered in the gamification process
38. Hisham and Sulaiman (2017)	Onboarding phase, Rewards, Leaderboard	Not specified
39. Jianu and Vasilateanu (2017)	Experience points, Levels, Ranks, Challenges, Instant feedback	Not specified

By analyzing the game elements listed in Table 2, we noticed that PBL elements (points, badges, and leaderboards), levels, and feedback are the most commonly used elements for gamifying e-learning systems in higher education. This is in line with other reviews’ findings, e.g. (Dichev & Dicheva, 2017).

Furthermore, in response to what (Dichev & Dicheva, 2017) stated about the fact that gamification with “deeper game elements” (Enders, 2013) by incorporating game design principles involving game mechanics and dynamics such as challenges, choice, low-risk failure, role-play or narrative is still scarce, we noted in our systematic literature review that recent studies explore new game elements. Indeed, among the 39 studies analyzed in Table 2, there are 20 primary studies that used “deeper game elements” (Enders, 2013) like challenges and storytelling (narrative). Among these, challenges are the most popular ones.

In Seaborn and Fels (2015), the authors noted that till 2015, the majority of applied research on gamification was not grounded in theory and did not use gamification frameworks in the design of the system under study. Likewise, in this systematic review, by analyzing the 39 empirical studies listed in Table 2, we noticed that most studies were not underpinned by gamification theories. This is in line with the findings of other recent studies, such as van Gaalen et al. (2021) and Kalogiannakis et al. (2021). Indeed, of the 39 primary studies analyzed in our systematic review, only nine papers (Smith, 2017; Kyewski & Krämer, 2018; Pilkington, 2018; Tsay et al., 2018; van Roy & Zaman, 2019; De-Marcos et al., 2020; Facey-Shaw et al., 2020; Sanchez et al., 2020; Dikcius et al., 2021) adapted theoretical approaches and used them as gamification approaches. These are a set of social and motivational theories resumed in a variety of six different theories, namely: self-determination theory-SDT, Social comparison theory, social exchange theory-SET, cognitive evaluation theory-CET, situated motivational affordance theory, theory of gamified learning (Landers, 2014) and user-centered design (Nicholson, 2012). Self-determination theory is considerably the most popular one. These findings are correlated with other reviews’ findings such as Zainuddin et al. (2020) and Kalogiannakis et al. (2021). Only two other primary studies Marín et al. (2019) and Dias (2017) used existing theoretical gamification frameworks to build their gamified e-learning systems. For the remaining papers, some built their owngamification design based on guidelines from the literature whereas others did not cite any theory. Hence, we notice that this distribution is in line with (Alhammad & Moreno, 2018)’s review findings regarding the use of four different categories of gamification approaches in primary studies, namely, papers that followed existing gamification frameworks, papers that adapted motivational theories to their needs, papers that built their own approach, and finally, those that didn’t follow any specific approach. We also noticed that motivational theories are the most frequently used approach, as noted in Ozdamli (2018).

#### **RQ2**

How these game elements are combined?

For this research question, we sought to identify how game elements are combined in gamified learning systems in higher education. Previous studies have not fully explored this point except the paper (Dichev & Dicheva, 2017). By analyzing the different empirical studies involved in this systematic literature review (listed in Table 2), we noticed the lack of detailed information about how instructors and designers combined different game elements. Indeed, in all reviewed papers, the authors listed only the game elements employed to gamify their learning systems. In addition, no study provided any justification of the choice made about the sets of game elements to use, nor the way they combined them in the gamified learning systems.

In the reviewed collection, five studies employed one single game element (Coleman, 2018; Garnett & Button, 2018; Kyewski & Krämer, 2018; Facey-Shaw et al., 2020; Dikcius et al., 2021), three other studies gamified systems using two game elements (Fajiculay et al., 2017; Smith, 2017; Donnermann et al., 2021), five other studies used three game elements (Hisham & Sulaiman, 2017; Kasinathan et al., 2018; Romero-Rodriguez et al., 2019; Khaleel et al., 2020; Sanchez et al., 2020) while the remaining ones used more than three elements.

This happens due to the lack of studies that provide clear guidelines and justifications for the combination of game elements (Toda et al., 2020).

#### **RQ3**

Which gamification design approaches are available in the literature?

In this section, we will approach **RQ3**. We first synthesize the current literature on gamification approaches in a general context. Then, we present a set of gamification approaches for general learning systems. Finally, we list a set of approaches proposed specifically for higher education within e-learning environments. We briefly described each approach in the table below (Table 3).

**Table 3:** Gamification approaches

Paper	Description of the approach
*General approaches*	
1. Deterding et al. (2011a, 2011b)	This research study founded the **MDA** model which based on elements of mechanics, dynamics, and aesthetics
2. Zichermann and Cunningham (2011)	Zichermann and Cunningham (2011) complemented the MDA model with other game elements such as: challenge, imagination, curiosity and control
3. Werbach and Hunter (2012)	A gamification model for gamified system development called the **6D** model refering to its six interrelated steps beginning with the letter D: define business objectives, delineate target behaviors, describe your players, devise activity loops, don't forget the fun, deploy appropriate tools
4. Nicholson (2012)	A user-Centered Theoretical Framework for designing Gamification
5. Chou (2015)	A gamification framework called **Octalysis** which is based on eight motivational drivers arranged in an octagonal shape. The elements of the Octalysis model that are in the right part represent are related to intrinsic motivation, as opposed to the elements on the left side, which relate to extrinsic motivation (Bernik, 2021). The elements at the top of the system are considered to be positive motivators that encourage the improvement of knowledge and skills through meaning and various incentives, whereas the elements at the bottom of the system are considered negative motivators that encourage bad emotion and should be minimized when planning and implementing the system (Bernik, 2021)
6. Andrade et al. (2016)	A framework for intelligent gamification (**FIG**) structured in three layers: gamification layer, tutor layer and data layer. It is important to note that this model is not approaching the content side of gamification. In this sense the gamification in this framework is a layer independent of the pedagogical objectives proposed by the tutor. This model is based on the following steps: information gathering, operation, assessment and adaptation
7. Morschheuser et al. (2017)	A method for designing gamification was developed which is the antecedent version of the one proposed in Morschheuser, Hassan et al. (2018)
8. Morschheuser et al. (2018)	A comprehensive detailed method for developing gamified software with a set of design principles
9. van Roy and Zaman (2017)	This paper forms a guide for researchers, educators, designers, and software developers in fostering a promising future generation of gamified systems
10. Ryan and Deci (2000)	This paper provides a framework called SDT (self-determination theory) for examining human motivation through its focus on innate psychological needs (competence, autonomy and relatedness) and the environments fostering or undermining motivation
11. Hunicke et al. (2004)	This paper presents the MDA framework (standing for Mechanics, Dynamics, and Aesthetics) a formal approach developed and taught as part of the Game Design and Tuning Workshop at the Game Developers Conference, San Jose 2001–2004
12. Alcivar and Abad (2016)	A method for gamifying ERPs (enterprise resource planning systems) was suggested
13. Cechetti et al. (2019)	A gamification method for promoting engagement in user’s treatment with the use of health-related systems
14. Aparicio et al. (2012)	A method for analysis and application of gamification as a tool to assist the participation and motivation of people in carrying out various tasks and activities
15. Enders (2013)	Guidelines for the design of gamified eLearning (can be also applied for training employees in companies so it remains general) systems, using gaming elements like points, achievements, badges, leaderboards, levels and challenge
16. García et al. (2017)	The paper proposed a framework for gamification in software engineering. This framework is composed of the ontology, a methodology for guiding the process and a support gamification engine. In a case study a company used the framework to gamify the areas of project management, requirements, management and testing
*Gamification approaches for general learning systems*	
1. Simões et al. (2013)	A **social gamification** framework for K-6 learning platform which applies to students from 6 up to 12 years old (K-6). This framework allows teachers to create challenges tailored to students’ level of knowledge; set up different ways to achieve an objective by creating multiple intermediate goals; provide feedback or immediate feedback that allows progress to a new task; to the proper game mechanics to the activities; consider failure as a part of the learning process; enable students to assume different identities and roles; enable recognition of the students’ progress by peers, teachers and parents; and use competition to promote valuable behaviours
2. Kim and Lee (2015)	This study proposed a Dynamical Model for structural and content Gamification of Learning (**DMGL**) after reviewing and comparing different models: Game Design Features (**GDF**), Key Characteristics of a Learning Game (**KCLG**), **ACRS** Model and **MDA** Framework to define four main factors of the proposed model, namely, challenge, fantasy, control and curiosity. In the DGML model, **control** is considered as the core characteristic. The relation between other factors is defined by authors: **curiosity** needs to be higher than the challenge. With time, motivation will be reduced, therefore, the ratio of the Challenge and the **fantasy** has efficient range and the proportion between them must be maintained in order to take the advantage of the educational effectiveness
3. Kapp (2012)	A book that provides broad guidelines for effective gamifying of learning and instruction
4. Llorens-Largo et al. (2016)	This paper provides lessons learned from a broad experience in using games and gamification in learning, and after several years of continuous feedback from students, on how to approach the task of gamification
5. Wendy Hsin-Yuan Huang (2013)	This paper is a report that represents a practitioner’s guide to gamification of learning programs
6. Wongso et al. (2014)	Proposed a conceptual framework design, based on Web 2.0 technology and gamification. The authors offered a guideline for implementing gamification and Web 2.0 technology in e-learning systems. Their framework includes the phases of analysis, design, development, implementation and evaluation
7. Böckle et al. (2018)	A design framework for developing adaptive gamification applications
8. Knutas et al. (2019)	A design process was proposed which is based on machine learning algorithm and personalized content selection.The process is based on Deterding’s framework for gameful design. The paper states that their novel contribution is demonstrating how both a personalization strategy and an algorithm creation process can be used to augment existing design processes, with the algorithm allowing automating the choice of personalization strategies and tasks. In this framework, there are seven design steps: 1. Define gamification strategy 2. Research 3. Select personalization strategies 4. Synthesis 5. Ideation 6. Distill rules into an algorithm 7. Rapid prototyping
9. Bennani et al. (2021)	An approach was proposed which is a **personalized** gamification model based on ontologies. Focusing on the online process of the approach, three activities are included in the process, namely, data collection (to capture explicit data that consists of students’ information and implicit data by proposing tests to students: player type test, intelligence type test, level test), data exploration (this sub-process is composed of Knowledge representation, Student profiling and Adaptation Recommender) and data reasoning
10. Rivera and Garden (2021)	A new Gamification Framework for Student Engagement was created and implemented allowing practitioners to systematically apply game attribute(s) to a learning experience to implement gamification for purposeful impact on student engagement outcomes in higher education
11. Duggal et al. (2021)	An intelligent open-ended (irrespective of course and the program being studied) gamified framework based on machine learning
12. Zhao et al. (2022)	An innovative gamification framework, called the NEWTON-enhanced gamification model (N-EGM), which was designed as part of the European Horizon 2020 project NEWTON
13. Lavoué et al. (2019)	A design process for adapting gaming features to learners’ player types based on a player model inspired from existing player typologies (this study used the BrainHex typology) and types of gamification elements. The model functions using the principle of recommender systems, by estimating the preference for a feature by a weighted sum of personality traits. Concretely, The model is based on matrix factorization of the matrix representing users’ profiles and the one representing the way in which gaming features match given player types
14. Park et al. (2019)	A design science framework which includes five iterative stages: 1. Problem definition, 2. Identification of desired outcomes, 3. Gamification design, 4. System development, 5. Evaluation. This model relies on Malone's theory of intrinsically motivating instruction and defines three categories of motivational drivers that are relevant to learning: challenge, curiosity and fantasy. This framework was implemented in practice to create the GAMESIT environment, a gamified system for information technology training
15. Zaric et al. (2020)	A framework for the design of a gamified personalized learning environment called **PeGam** for: Personalized Gamification Design Model. This framework is based on user-centered gamification **(**Nicholson, 2012**)**, and suggests five conceptual elements to be considered: the purpose of personalization, personalization criteria, personalized game elements, personalized gamified intervention, intervention evaluation
16. Toda et al. (2020)	A design method for gamifying learning systems using the Design Sprint method and by instantiating the taxonomy proposed by (Toda, Oliveira et al. 2019)
17. Towongpaichayont (2021)	A guideline for designing classroom gamification is proposed which includes: 1. identify the pillar roles of the classroom 2. identify expected pain points in the classroom, 3. identify expected overall aesthetics and the purposes of including gamification into the classroom, 4. design mechanics in the class, 5. pick the right elements and tools for the classroom, and 6. Iterative monitoring and adjustments
18. Rodríguez et al. (2022)	A dynamic adaptive gamification method which takes players’ profiles as initial information and also considers how these profiles change over time based on users’ interactions and opinions
*Gamification approaches for e-learning systems in higher education*	
1. Urh et al. (2015)	A model for introduction of **gamification** into **e-learning** environments in **higher education** that consists of the following main elements: management of e-learning, important factors in e-learning, elements of user experience, phases of development (analysis, planning, development, implementation, and evaluation), game mechanics, game dynamics, gamification elements in e-learning and their effects on students. This study considers the management of e-learning as an important part of the model. The proposed model considers multiple elements for user experience: project management, user research, usability evaluation, information architecture, user interface design, visual design, interaction design, content strategy, accessibility and web analytics.
2. Mi et al. (2018)	A systematic incentive model was proposed for motivating students to learn code readability in software engineering, with the combination of both intrinsic (crowdsourcing) and extrinsic (GDEs: points, badges, leaderboards) motivators. This method was implemented as an online platform GamiCRS for students to learn code readability
3. Huang and Hew (2018)	A gamification design model was proposed based on aspects derived from five motivation **theories**, namely, flow theory, goal-setting theory, social comparison theory, self-determination theory and behavior reinforcement theory. This model is called the **GAFCC** design model to goal-access-feedback-challenge-collaboration. In order to implement the model in practice, the paper recommends following the five-stage gamification design procedure of examine, decide, match, launch, and evaluate.
4. Carreño (2018)	A framework for the design of personalized gamification services. The framework, called **FRAGGLE** (FRamework for AGile Gamification of personalized Learning Experiences) is based on the use of the Agile methodologies to obtain a fast design ready for testing and being able to iterate. This framework is structured in four phases: 1. Declaration, 2. Creation, 3. Execution, 4. Learning. In the **declaration** phase which serves mainly for information gathering, four main key concepts must be sequentially declared: problems, causes (of the previously identified problems; it can be carried out by the “Five Why” technique), user stories (description of the desired outcomes which consist of objectives) and acceptance tests (expected concrete behaviors in the form of when…, then…). At the **creation** stage, the appropriate design components are defined: players, game mechanics, stages (discovery, on-boarding, mid-game, and endgame), actions (description of desired and undesired performances) and triggers (to give response to user actions). In the **execution** phase, the learner interacts with the developed system. Information about behavior and user’ interactions must be tracked in order to promote the developed system in the future versions. The **learning** phase serves to test the effectiveness of the developed activities
5. Kamunya et al. (2020)	An adaptive gamification model was developed to guide and implement adaptivity within e-learning platforms. Its key elements are: The Adaptive gamification engine, Management of the E-learning platform, Adaptive game elements techniques and dynamics and adapted gamified course. This work is based on a previous proposed model in the literature **(Urh, Vukovic et al. **2015**),** with a focus on learner individuality
6. Legaki et al. (2020)	A gamification approach was developed, called: Horses for Courses based on guidelines offered by prior studies. The study is scenario-based, and four gamification affordances were identified from the literature to be implemented in the Horses for Courses application namely, points, levels, leaderboards, and challenges
7. Alsubhi and Sahari (2020)	A conceptual gamification framework to guide developers in the process of incorporating game elements into LMS systems; The framework consists of three components: game elements or gamification components; learning activities; and student engagement components. Game elements, which influence **learning activities**, are thus grouped and subsequently mapped to the corresponding activities
8. Winanti et al. (2020)	A gamification framework for **higher education**, especially for programming language courses. The proposed framework contains the main activities: 1. Participant identification. 2. Objective identification. 3. Implementation. 4. Learning evaluation;
9. Bencsik et al. (2021)	A gamification model was proposed based on literature review, containing two main phases: Phase 1: planning the process: The logical process of this model contained 4 main steps: Familiarization, Acclimatization, Immersion, and testing. Phase 2: ‘persona generation’: describe participant motivation
10. Fajri et al. (2021)	A gamification model was proposed to be used in blended learning in higher education, using 2 mechanisms: feedback mechanism (points, badges, rewards) and presentation mechanism (Progress bar, Leaderboard)
11. Alsubhi et al. (2021)	An engagement framework for guiding developers when **gamifying e-learning** systems within the **higher education** context. This work is based on the previous version **(**Alsubhi & Sahari, 2020**)**
12. Yamani (2021)	A conceptual framework for gamification integration in eLearning systems based on the instructional design (ID) model. The stages of this framework are managerial process, analysis, design, development, implementation, evaluation
13. Al Ghawail et al. (2021)	A gamification model in the e-learning environment in the Libyan higher education context, presented in terms of ADDIE, these five key elements of the ADDIE model include: Analysis, Design, Development, Implementation, and Evaluation
14. Sofiadin and Azuddin (2021)	A gamification framework for higher education to assist institutions in designing a gamified e-learning that supports and enables a sustainable education. The key elements of this framework are teaching and learning principles, technology, applications and security & ethics
15. Júnior and Farias (2021)	A Quality Model for Gamified Software Modeling Learning (example: UML modeling), called ModelGame. It serves as a reference framework intended for **higher education** institutions teaching software modeling
16. Bernik (2021)	A conceptual model called eRIOOS intended to **higher education** for gamifying educational **e-courses** at higher education institutions. The aim of this research was to standardize the gamification elements that can be used in educational e-courses at higher education institutions
17. de la Peña et al. (2021)	A gamification model for **university-level distance learning**, where game choice is based on skill type and the learning objectives to be attained. The proposed model is composed of the following steps: 1. Choice of the course to be gamified in the subject 2. Set the parameters of the course 3. Choice of gamification technique 4. Development of the course 5. Roll out 6. Results and validation 7. Lessons learned

In the table above, we investigated a total of 51 gamification approaches in three different contexts. The first set of approaches (the first 16 rows of Table 3) was designed for general use, i.e., for all contexts such as learning, health, marketing and entrepreneurship. While the second set of approaches (the next 18 rows of Table 3) targeted general learning contexts, i.e., without any restriction on educational level. Finally, the third set of approaches (the last 17 rows of Table 3) was intended to be applied in a specific context, namely digital higher education.

Given our review’s main interest in e-learning in higher education, we will classify the last 17 approaches of Table 3, which correspond to those designed for e-learning systems in higher education, into several classes based on different relevant criteria that we will detail below. The paper (Saggah et al., 2020) proposes categorizing gamification design frameworks into three categories: scenario-based, high-level approach, and Gamification elements guidance. Inspired by this categorization, we propose our categorization, which will be used to classify the different gamification approaches in e-learning in higher education. A description of each category is given in what follows, and our classification results are shown in Table 4.

- Level of detail
  - *High-level approach* This group categorizes papers that provide an overview of the design process that serves as a general high-level guideline containing the global phases without detailing which game elements to use and how to implement them.
  - *Gamification elements guidance* This group categorizes papers that provide a conceptualization of the gamification elements that can be used in educational environments. These studies can include implementation guidance.
  - *Scenario based* This group categorizes papers that provide a descriptive outline of the design process. In other words, these papers propose gamification approaches by describing their application through real empirical studies experimented in real learning environments.
- *Type from student perspective (adaptive gamification/one size fits all gamification)* Adaptive gamification considers that users have different motivations, so it consists of personalizing learning experiences according to each learner profile. Whereas ‘one size fits all’ gamification uses the same gamified system (gamification elements, rules, etc.) for all learners. For ease of use, we will use ‘A’ character for adaptive approaches and x for ‘one size fits all’ ones.

**Table 4:** Classification of gamification approaches (context of e-learning in higher education)

Paper	Level of detail			Type	Profundity	Validation
	high-level approach	Scenario based approach	Gamification elements guidance			
1. Urh et al. (2015)			x	x	x	
2. Mi et al. (2018)		x		x	x	
3. Huang and Hew (2018)			x	x	x	x
4. Carreño (2018)	x			A	x	
5. Kamunya et al. (2020)			x	A	x	
6. Legaki et al. (2020)		x		x	x	
7. Alsubhi and Sahari (2020)			x	x	x	
8. Winanti et al. (2020)			x	x	x	
9. Bencsik et al. (2021)		x		x	x	
10. Fajri et al. (2021)		x		x	x	
11. Alsubhi et al. (2021)			x	x	x	X (With experts)
12. Yamani (2021)			x	x	x	
13. Al Ghawail et al. (2021)		x		x	x	
14. Sofiadin and Azuddin (2021)			x	x	x	
15. Júnior and Farias (2021)			x	x	x	x
16. Bernik (2021)		x		x	x	
17. de la Peña et al. (2021)	x			x	x	x
- *Profundity from pedagogical perspective (structural gamification versus content gamification)* structural gamification refers to the application of game design elements to motivate the learner through an instructional content without changing it (Garone & Nesteriuk, 2019). It can be made by using clear goals, rewards for achievements, progression system and status, challenge and feedback (Garone & Nesteriuk, 2019). Content gamification is the application of elements, mechanics and game thinking to make the content more game-like (Garone & Nesteriuk, 2019). It is a one-time structure created only for a specific content or learning objectives and hence cannot be reused for any content (Sanal, 2019). Garone and Nesteriuk (2019) states that elements that can be used in content gamification are story and narrative; challenge, curiosity and exploration; characters and avatars; interactivity, feedback and freedom to fail (Kapp, 2014). According to Kapp (2014), the combination of both structural and content gamification, is the most effective way to build high engaging and motivating environments. For ease of use, we will use ‘C’ character for content approaches and x for structural ones.
- *Validation* This group categorizes papers that provided a validation of the proposed approach through empirical evidence showing its application to e-learning systems in higher education.

Table 4 represents the results of our classification of gamification approaches in the context of e-learning in higher education. Regarding the level of detail, we noticed that most of the analyzed approaches (with a number of 9 out of a total of 17) are of the type of gamification elements guidance (Urh et al., 2015; Huang & Hew, 2018; Alsubhi & Sahari, 2020; Kamunya et al., 2020; Winanti et al., 2020; Alsubhi et al., 2021; Júnior & Farias, 2021; Sofiadin & Azuddin, 2021; Yamani, 2021). This number is followed by a number of 5 approaches of type scenario based (Mi et al., 2018; Legaki et al., 2020; Al Ghawail et al., 2021; Bencsik et al., 2021; Fajri et al., 2021), and finally, only 2 approaches are categorized as high-level approaches (Carreño, 2018; de la Peña et al., 2021). It is worth saying that scenario-based approaches are, in most cases, the most difficult to reproduce in other educational environments, as they are very specific, and each environment has its own characteristics. In contrast, high-level approaches are more general and need to be tailored according to the context. Finally, gamification elements guidance approaches can strongly help implement gamified learning environments as they provide a handy catalog of elements that can be injected easily into learning environments.

Furthermore, Table 4 shows that most of the suggested design approaches in the literature are not empirically explored (for example, by using a control and comparing gamified and non-gamified systems). Indeed, of the 17 gamification approaches in the context of e-learning in higher education analyzed, only four approaches have been applied and evaluated by empirical evidence (Huang & Hew, 2018; Alsubhi et al., 2021; de la Peña et al., 2021; Júnior & Farias, 2021). Among those four studies, one work was validated with experts (Alsubhi et al., 2021).

Moreover, Table 4 shows that of the 17 gamification approaches proposed for application to online learning systems in the context of higher education, two approaches (Carreño, 2018; Kamunya et al., 2020) fall into the category of adaptive gamification. This shows the trendy nature of personalization in higher education. Finally, Table 4 shows that the 17 approaches that have been proposed to gamify online learning systems in higher education focus solely on structured gamification, neglecting the content side of online learning systems.

## Discussion and limitations

Through this systematic review, we identified several papers on the gamification of e-learning in the higher education context. In recent years, the research on gamification in e-learning has been getting traction, and the number of research articles and systematic reviews of research articles is increasing. As a summary of the existing approaches of gamification in e-learning in higher education, we notice the following points:

### Gamification of e-learning in higher education: a trending area of research

**Fig. 2:**
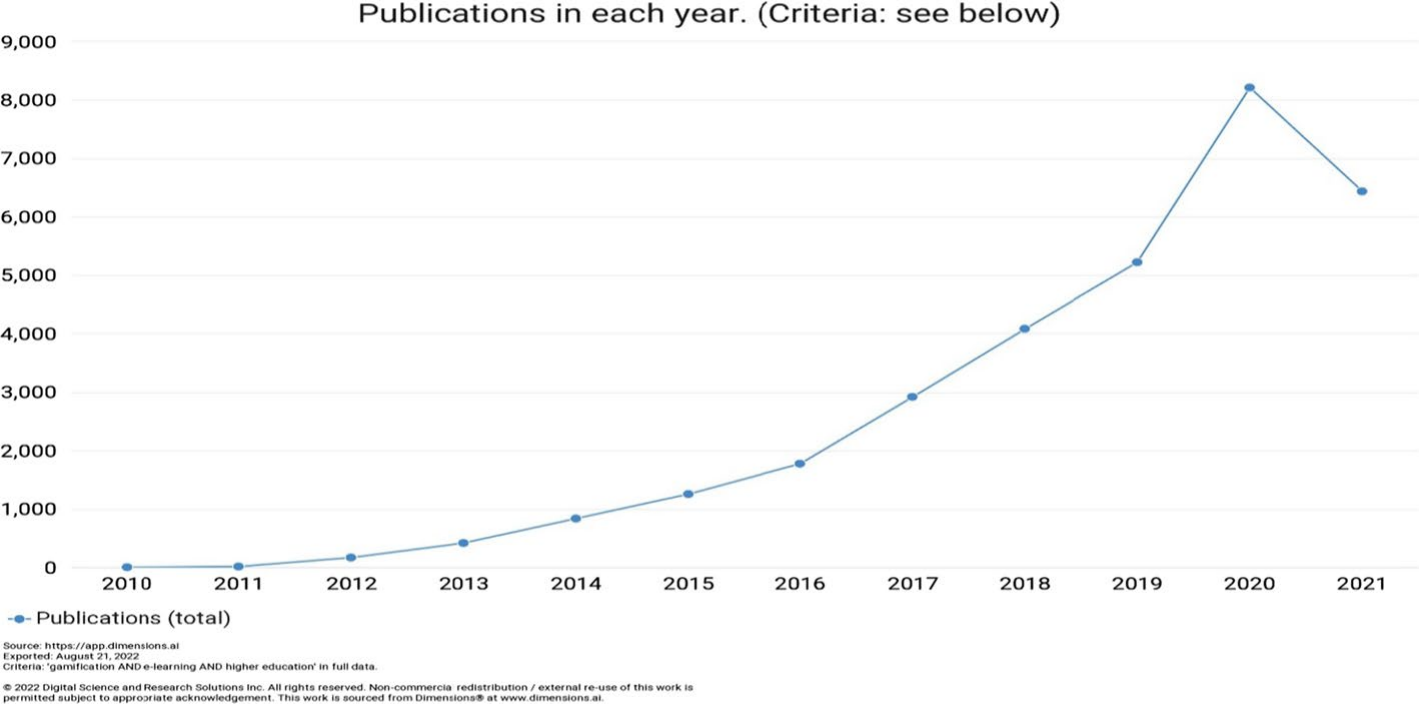
Number of publications per year

The systematic review ﻿showed that gamification of learning systems is nowadays a hot topic, and research in this field is growing rapidly as well as for e-learning in higher education context, as it is shown by Fig. 2.

### Gamification design gaps and tendencies

In general, gamification theory helps in training and shaping participant behavior, however, in our systematic literature review, we observed from RQ1 that the majority of applied research on gamification is not grounded in theory and did not use gamification frameworks in the design of the learning system under study. This highlights the fact that there is a real gap between theoretical and applied work on gamification. One reason may be that existing approaches are very theoretical and cannot strongly assist designers and practitioners when gamifying learning systems, as pointed out by Toda et al. (2020). This also explains our results to the second research question RQ2 regarding the lack of detail on the combination of game elements used in the experimental studies and the motivation behind choosing specific game elements over others.

To better understand this phenomenon and to find a rationale for this lack of using theory and, thus, the lack of logic behind the use of certain game elements over others and their random linking and combination in gamified learning systems, we addressed the research question RQ3. In the latter, we analyzed the gamification approaches available in the literature and classified them into different categories based on a variety of criteria. Our results revealed that the gamification elements guidance approaches that provide taxonomies of game elements that can be incorporated into learning systems constitute the majority of the approaches that have been proposed for application in online learning in higher education. Those did not provide the psychological and behavioral changes that correspond to each game element. Instead, the older gamification theory was based simply on the behavioral outcomes that come from using gamification and the motivational needs behind it and did not provide details on how to implement them or details on what elements to use.

Using appropriate game elements can lead to higher levels of user motivation, whereas inappropriate game elements can demotivate users (Hallifax et al., 2019a, 2019b). Thus, it is essential to choose the right combination of game elements that perfectly matches the desired behavior change. To do this, we must first explore the effect of each game element separately (Dichev & Dicheva, 2017). Thus, further studies are needed to improve our understanding of how individual game elements relate to behavioral and motivational outcomes so that we can identify their contribution in studies that mix multiple game elements (Dichev & Dicheva, 2017). An example of such study was provided in the health domain in the paper (Hervas et al., 2017). The latter proposed a taxonomy of gamification elements used in the domain of health by relating them to psychological fundamentals on behavioral changes, like Self-efficacy, Social influence, and Behavioral momentum. This work can facilitate researchers' empirical validation of gamification theory by building contexts and scenarios from ready-made taxonomies of gamification elements that target a specific behavioral outcome.

On the other hand, through our systematic literature review, we can see from RQ3 the recent emergence of data-driven approaches through machine learning techniques (Knutas et al., 2019; Duggal et al., 2021). These techniques help to create gamification designs suitable for the gamified context, especially when it comes to customizing the game elements to be incorporated into the final gamified system to the students' profiles.

In many learning environments, pedagogy assumes that all learners have homogeneous characteristics (Kamunya et al., 2020). However, Schöbel and Söllner (2016) argue that most gamification projects are not working because they are designed for a group of system users without considering the personal needs of each user. Hence the advantage of personalized training to the learner where all learners differ in preference, style and abilities with regard to the learning processes with or without technology mediation (Naik & Kamat, 2015). In this context, we noted the existence of two gamification approaches designed for online learning in higher education (Carreño, 2018; Kamunya et al., 2020). This is put into practice by tailoring the gamification elements to users' individual preferences. A recent related problem is the lack of adaptation of gamification to the content being gamified.

Another recent and relevant issue is the extreme lack of content gamification. Indeed, the motivational impact of certain game elements varies with the user activity or the domain of gamified systems (Hallifax et al., 2019a, 2019b). Therefore, there is a great need for further exploration and experimentation in this immature area to provide a gamified design to satisfy users’ preferences as well as the task at hand. In other words, personalization in gamification should extend to content, as it does with user profiles, for example, by applying machine learning techniques to tailor the choice of game elements to gamified content.

Another common study design issue illuminated by our review is the lack of validation of the proposed gamification approaches through statistical analyses. In addition, most applied research on the gamification of online learning systems in higher education has not explored the gamification frameworks suggested in the literature.

## Conclusion and future work

In this work, we conducted a review of the literature on gamification elements used in digital higher education, the way they are combined, and the different gamification approaches proposed in the literature to gamify learning systems. We analyzed a total of 90 papers to answer the three research questions formulated for this study.

This review identified points, badges, leaderboards, levels, feedback, and challenges as the most commonly used game elements in digital higher education. However, in terms of using gamification theory, our review found that the majority of applied gamification research is not theory-based and has not used gamification frameworks in the design of gamified learning systems. Although some experimental studies attempt to adapt psychological and educational theories available in the literature as gamification approaches, the resulting systems are not very clear, and there is no rationale for choosing certain game elements over others. Consequently, it can be concluded that these gamification approaches cannot strongly assist designers and practitioners in gamifying their learning systems. In addition, theoretical gamification approaches in e-learning in higher education should focus on understanding the effect of each single game design element and the behavioral changes that outcome from its use.

Moreover, based on the results of this review, we can observe the trend towards data-driven approaches through the use of machine learning techniques, especially in adaptive gamification approaches. This involves the adaptation of gamification elements to user profiles. On the other hand, although we have noticed the increasing use of gamification elements that are suitable for content gamification and make the content more game-like, such as storytelling and challenges, there is still a lack of gamification approaches that address content gamification. In fact, this is still an immature research area in gamification design in e-learning in higher education.  Future works should pay more attention to the pedagogical side of learning systems and the task under gamification. Apart from that, further research is required to compare theory-driven to data-driven gamification approaches, in terms of which one is the better or perhaps evaluate the effectiveness of a combination of the two, and go so far as to propose a hybrid gamification approach, which does not exist yet and might solve several gamification design issues.

Regarding future work, efforts should focus on building a holistic approach by considering all the aspects that constitute the environment. Among those,  personalization according to students’ profiles, gamified subject, educational context, learner’s culture, learner’s preferences, level, playing motivations and experience with games.

Finally, we have seen that most of the design approaches suggested in the literature are not empirically explored. Therefore, statistical analyses and comparative studies should be conducted to draw more robust and generalizable conclusions to validate the existing gamification approaches in the literature.

## Data Availability

All data generated or analyzed during this study are included in this published article.

## Appendix

**Table:**

Paper	Description
1. Romero-Rodriguez et al. (2019)	A mixed-quasi-experimental study where 12 gamified MOOC platforms were considered to analyze how the application of gamification strategies in MOOCs on energy sustainability affects participants’ commitment
2. Bernik et al. (2019)	The research was conducted in two phases: pilot study and main study; in both, two versions (gamified and non-gamified) of an e-module were taught and the same content was delivered. the goal was to examine the effects of using gamification on learning achievements
3. Facey-Shaw et al. (2020)	A quasi-experimental study that sought to address the extent to which badges had an effect on intrinsic motivation of Introductory Programming students
4. Bernik et al. (2017)	An experimental study on efficiency of applying gamified design into University's e-courses: 3D modeling and programming, conducted in two Croatian higher education institutions that included both full-time and part-time students
5. Guérard-Poirier et al. (2020)	A randomized controlled trial that aims to evaluate the efficacy and usability of web-based peer-learning for advanced suturing Techniques. An educational network for surgical education supported by gamification elements and GRS system (global rating scale) were used
6. Kasinathan et al. (2018)	A mobile application ‘Questionify’ was developed using C# and java languages, which was intended to students of Software Engineering course. In this paper, the application was described in detail and some elements of application design are explored in depth (database tables, interfaces, structure)
7. Kyewski and Krämer (2018)	An experimental study on the influence of badges on motivation, activity, and performance in an online learning course conducted during an online seminar at a German university over a period of one semester and Moodle platform was used. Students registered for the online course titled: “Basic psychological mechanisms of computer-mediated communication: learning and teaching”
8. Dikcius et al. (2021)	An experiment that sought to determine the effect of gamification rewards and social interactions on students in an online marketing course
9. Yildirim (2017)	An experimental study that aims to determine the effects of gamification-based teaching practices on student achievement and their attitudes toward lesson by gamifying a blended learning course ‘Teaching Principles and Methods’. The study's participants consist of sophomores in the Department of Elementary Mathematics Education at a state university in southern Turkey during the 2014–2015 academic years
10. Fajiculay et al. (2017)	The purpose of this article is to describe student perceptions of implementation of digital badges in a drug information and literature evaluation course. Two digital badges were developed: “Communication of Drug Information” and “Evaluation of Medical Literature”
11. Pilkington (2018)	This study explores promoting motivation in a distance education, in a third-year computer programming course via a gamified approach to improve coursework participation and student experience
12. Dichev and Dicheva (2017)	This study investigated how the use of meaningful gamification affects student learning, engagement, and affective outcomes in a short, 3-day blended learning research methods class using a combination of experimental and qualitative research methods
13. Khaleel et al. (2019)	This study aimed to measure the effectiveness and motivation level of using a gamification website for programming language learning for first year students. Quantitative research approach was used. The effectiveness of the gamification website was tested using a quasi-experiment. Student motivation was measured using ARCS motivation model
14. Pérez-López et al. (2017)	The aim of this paper is to describe an innovation experience in the university classroom via a gamification proposal. The assessment of the experience was obtained from anonymous narratives submitted by the students to Google Drive once the experience ended. These narratives were analyzed with the support NVivo10 software
15. Tsay et al. (2018)	This paper evaluated the use of gamification to facilitate a student-centered learning environment within an undergraduate year 2 Personal and Professional Development (PPD) course
16. Aşıksoy (2018)	In this study, a true experimental design was used. The study was conducted with 61 undergraduate students taking a Physics-2 course. The experimental group students learned in the gamified flipped classroom environment, while the control group students learned with the flipped classroom approach without a gamification strategy
17. Khaleel et al. (2020)	The main objective of this experimental study is to increase student engagement in learning programming subject, and also to measure the impact of game elements on student’s engagements. The study presented a use case diagram and active diagram for the overall process of designing the gamification website
18. Gunawan and Jupiter (2018)	This study aims to evaluate the effectiveness of gamification in e-learning. For this purpose, an educational website, www.bangsacerdas.com, was established. There are two parts of participants: the students who are directed to a learning system with gamification and the students who are enrolled in a learning system without gamification. During the process, the level of user engagement and the quality of learning are being evaluated in each group. The t-test
19. Bilgin and Gul (2020)	The aim of the study was to investigate the effect of gamification (online and face-to-face) on the attitudes of students towards working as small groups, the course, and their academic achievement. Edmodo was used as the gamified online platform
20. Buckley and Doyle (2017)	This research examines the impact that different learning styles and personality traits have on students'; (1) perceptions of, (2) engagement with and, (3) overall performance in a gamified learning intervention developed using a prediction market. The study evidences a range of responses to gamification based upon individual learning styles and personality traits
21. Sanchez et al. (2020)	This paper applies the theory of gamified learning and extends research exploring the benefits of gamification on student learning through the testing effect. In a quasi-experimental design, university students (N = 473) prepared for three tests using traditional quizzes (i.e., a question, four response options) or gamified online quizzes
22. Asiksoy and Canbolat (2021)	In this study, a Gamified Flipped Classroom (GFC) environment proposes a solution to the issue of lack of participation of the students in online activities within flipped learning systems. A true-experimental design was used in the study and the effects of teaching in this environment on students’ online behaviors and achievements were investigated
23. Adams and Du Preez (2021)	This study applied a design-based research approach which offers a contextually sensitive, theoretically driven approach to the design and refinement of educational interventions. Through iterative implementations and qualitative data collection, over a 2-year period, the process and outcome of gamifying the learning activities in an Industrial Psychology module to facilitate student engagement were reported
24. Garnett and Button (2018)	This paper reflects one part of a whole study using gamification techniques to motivate first-year nursing students to prepare for bioscience practical classes. The teaching topic used for this study incorporated digital badges into the online learning platform (Moodle) to be offered as a reward for completing pre-class activities
25. Castro and Gonçalves (2018)	An exploratory, applied, and technological innovation research, with a qualitative and quantitative approach, developed at a university in the southern region of Brasilia between February and November 2016. The aims of this study was to investigate whether the course offer with elements of gamification contributes to the formation of competences in Informatics in Nursing, and evaluate it based on teaching and learning criteria and content structure
26. Coleman (2018)	This action research was conducted to guide the implementation of a badging system at Maranatha Baptist University. It seeks to determine how to best optimize a co-curricular digital badging system for maximum student engagement through a combination of extrinsic and intrinsic motivators
27. Ropero-Padilla et al. (2021)	The aim of this study was to explore nursing students’ experiences and perceptions of the use of game elements in two full-nursing subjects using a blended-learning teaching strategy. A blended-learning teaching approach with game elements was developed for two full-undergraduate nursing subjects. Focus groups using a semi-structured interview protocol were conducted after delivering the teaching content
28. Gündüz and Akkoyunlu (2020)	This study aimed to investigate the effect of the use of gamification in the online environment of flipped learning to determine whether it will increase interaction data, participation, and achievement. A mixed-methods sequential explanatory design was used, which implies collecting and analyzing quantitative and then qualitative data. In the online learning environment of the experimental group gamification was integrated
29. Milenković et al. (2019)	This paper investigates the use of gamification for educating engineers in the field of biometrics. A learning platform with gamification elements was developed for the course of biometric technologies, held at the University of Belgrade
30. Donath et al. (2020)	This paper is a conceptual approach to education for sustainable development using an e-learning platform. The article presents a conceptual design of the learner’s journey and a mapping from gamification concepts to Moodle LMS elements
31. Pakinee and Puritat (2021)	This study presents an applied gamification concept to e-learning focusing on improving engagement of the various types of personalities of undergraduate students in ERP courses. The gamification design was developed by implementing the pros and cons of each game element to compromise the overall performance of students
32. van Roy and Zaman (2019)	This article aims at gaining an in-depth understanding of the power of gamification as shaping motivation based on the principles of basic psychological need satisfaction derived from Self-Determination Theory. This study turned throughout a 15-week university master course where students voluntary interacted with a gamified google + community platform
33. Ahmed and Asiksoy (2021)	This study investigated the effects of the Gamified Flipped Learning (GFL) method on students’ physics self-efficacy and innovation skills in a virtual physics laboratory course. The study was carried out with true experimental design and the participants were a total of 70 first-year engineering students, which were randomly divided into two groups. The experimental group was trained with the GFL method, the control group was trained with Classical Flipped Learning (CFL) method
34. Marín et al. (2019)	The main goal of this article is to obtain empirical evidence on the improvement of students’ learning performance when using UDPiler in comparison to a non-gamified compiler. A quasi-experiment was performed with two groups of first-year engineering students at Diego Portales University in Chile, using a non-gamified compiler and a gamified platform, respectively
35. De-Marcos et al. (2020)	This paper analyzes the effects of gamification in the social network of a massive online course. An educational social-networking platform gathered information about the contributions of participants and about the social networks that were formed during the course
36. Donnermann et al. (2021)	This paper describes the creation process of a learning environment for students in higher education and implemented additions (social robot and gamification) based on guidelines for gamification in learning scenarios, and research on pedagogical agent
37. Dias (2017)	An empirical study comparing the experiences of students taking a gamified course with those of students taking the non-gamified version measured over four semesters of an undergraduate operations research class taken by 150 first-year management students is presented
38. Smith (2017)	A quasi-experimental study that gamified three modules in Statics course, intended to undergraduate students. The gamified version of the modules were compared to its counterpart of non-gamified version, by assessing students’ attitudes towards the course
39. Hisham and Sulaiman (2017)	This study describes the process of applying gamification on online courses platform. An experiment was conducted to test the effects of the gamified platform on students’ engagement, involving a total number of 50 students
40. Jianu and Vasilateanu (2017)	This study presents the implementation of an adaptive gamified system for learning. The creators of the system sought to make adaptive by scaling and reuse questions, i.e., adjusting the level of questions according to student’s level. Questions used are of two types: theoretical and reasoning

## References

[CR1] Adams, S. P., & Du Preez, R. (2021). Supporting student engagement through the gamification of learning activities: A design-based research approach. *Technology, Knowledge and Learning,**27*, 119–138.

[CR2] Ahmed, H. D., & Asiksoy, G. (2021). The effects of gamified flipped learning method on student’s innovation skills, self-efficacy towards virtual physics lab course and perceptions. *Sustainability (switzerland),**13*(18), 10163.

[CR3] Al Ghawail, E. A., Yahia, S. B., et al. (2021). *Gamification model for developing E-learning in Libyan Higher Education. Smart education and e-learning 2021* (pp. 97–110). Springer.

[CR4] Alcivar, I., & Abad, A. (2016). Design and evaluation of a gamified system for ERP training. *Computers in Human Behavior,**58*, 109–118.

[CR5] Alhammad, M. M., & Moreno, A. M. (2018). Gamification in software engineering education: A systematic mapping. *Journal of Systems and Software,**141*, 131–150.

[CR6] Allen, M. W. M. W. (2007). *Designing successful e-learning: Forget what you know about instructional design and do something interesting*. Wiley.

[CR7] Alsubhi, M., Ashaari, N., et al. (2021). Design and evaluation of an engagement framework for e-learning gamification. *International Journal of Advanced Computer Science and Applications*, *12*.

[CR8] Alsubhi, M. A., & Sahari, N. (2020). A conceptual engagement framework for gamified E-learning platform activities. *International Journal of Emerging Technologies in Learning,**15*(22), 4–23.

[CR9] Andrade, F. R. H., Mizoguchi, R., et al. (2016). *The bright and dark sides of gamification*. Springer.

[CR10] Aparicio, A. F., Vela, F. L. G., et al. (2012). Analysis and application of gamification. In *Proceedings of the 13th international conference on Interacción Persona-Ordenador*. Elche, Spain: Association for Computing Machinery. Article 17.

[CR11] Aşıksoy, G. (2018). The effects of the gamified flipped classroom environment (GFCE) on students’ motivation, learning achievements and perception in a physics course. *Quality and Quantity,**52*, 129–145.

[CR12] Asiksoy, G., & Canbolat, S. (2021). The effects of the gamified flipped classroom method on petroleum engineering students’ pre-class online behavioural engagement and achievement. *International Journal of Engineering Pedagogy,**11*(5), 19–36.

[CR13] Bencsik, A., Mezeiova, A., et al. (2021). Gamification in higher education (case study on a management subject). *International Journal of Learning, Teaching and Educational Research,**20*(5), 211–231.

[CR14] Bennani, S., Maalel, A., et al. (2021). Towards an adaptive gamification model based on ontologies. In *2021 IEEE/ACS 18th international conference on computer systems and applications (AICCSA)*.

[CR15] Bernik, A. (2021). Gamification framework for E-learning systems in higher education. *Tehnički Glasnik,**15*(2), 184–190.

[CR16] Bernik, A., Radošević, D., et al. (2017). Research on efficiency of applying gamified design into University’s e-courses: 3D modeling and programming. *Journal of Computer Science,**13*(12), 718–727.

[CR17] Bernik, A., Radošević, D., et al. (2019). Achievements and usage of learning materials in computer science hybrid courses. *Journal of Computer Science,**15*(4), 489–498.

[CR18] Böckle, M., Micheel, I., et al. (2018). A design framework for adaptive gamification applications.

[CR19] Buckley, P., & Doyle, E. (2017). Individualising gamification: An investigation of the impact of learning styles and personality traits on the efficacy of gamification using a prediction market. *Computers and Education,**106*, 43–55.

[CR20] Carreño, A. M. (2018). A framework for agile design of personalized gamification services.

[CR21] Castro, T. C., & Gonçalves, L. S. (2018). The use of gamification to teach in the nursing field. *Revista Brasileira De Enfermagem,**71*(3), 1038–1045. 29924165 10.1590/0034-7167-2017-0023

[CR22] Cechetti, N. P., Bellei, E. A., et al. (2019). Developing and implementing a gamification method to improve user engagement: A case study with an m-Health application for hypertension monitoring. *Telematics Informatics,**41*, 126–138.

[CR23] Chou, Y. K. (2015). *Actionable gamification: Beyond points, badges, and leaderboards*. Createspace Independent Publishing Platform.

[CR24] Coleman, J. D. (2018). Engaging undergraduate students in a co-curricular digital badging platform. *Education and Information Technologies,**23*(1), 211–224.

[CR25] de la Peña, D., Lizcano, D., et al. (2021). Learning through play: Gamification model in university-level distance learning. *Entertainment Computing,**39*, 100430.

[CR26] De-Marcos, L., Garcia-Cabot, A., et al. (2020). Gamifying massive online courses: Effects on the social networks and course completion rates. *Applied Sciences (switzerland),**10*(20), 1–17.

[CR27] Deterding, S., Dixon, D., et al. (2011b). From game design elements to gamefulness: Defining gamification.

[CR28] Deterding, S., Dixon, D., et al. (2011a). From game design elements to gamefulness: Defining "gamification". In *Proceedings of the 15th international academic mindtrek conference: envisioning future media environments* (pp. 9–15). Tampere, Finland: Association for Computing Machinery.

[CR29] Deterding, S. (2012). Gamification: Designing for motivation. *Interactions,**19*(4), 14–17.

[CR30] Dias, J. (2017). Teaching operations research to undergraduate management students: The role of gamification. *The International Journal of Management Education,**15*(1), 98–111.

[CR31] Dichev, C., & Dicheva, D. (2017). Gamifying education: What is known, what is believed and what remains uncertain: A critical review. *International Journal of Educational Technology in Higher Education,**14*(1), 9.

[CR32] Dicheva, D., Dichev, C., et al. (2015). Gamification in education: A systematic mapping study. *Educational Technology & Society,**18*, 75–88.

[CR33] Dikcius, V., Urbonavicius, S., et al. (2021). Learning marketing online: The role of social interactions and gamification rewards. *Journal of Marketing Education,**43*(2), 159–173.

[CR34] Donath, L., Mircea, G., et al. (2020). E-learning platforms as leverage for education for sustainable development. *European Journal of Sustainable Development,**9*(2), 1–19.

[CR35] Donnermann, M., Lein, M., et al. (2021). Social robots and gamification for technology supported learning: An empirical study on engagement and motivation. *Computers in Human Behavior,**121*, 106792.

[CR36] Duggal, K., Gupta, L. R., et al. (2021). Gamification and machine learning inspired approach for classroom engagement and learning. *Mathematical Problems in Engineering,**2021*, 9922775.

[CR37] Enders, B. (2013). *GAMIFICATION, GAMES, AND LEARNING: What managers and practitioners need to know*. The E-learning Guild.

[CR38] Facey-Shaw, L., Specht, M., et al. (2020). Do badges affect intrinsic motivation in introductory programming students? *Simulation and Gaming,**51*(1), 33–54.

[CR39] Fajiculay, J. R., Parikh, B. T., et al. (2017). Student perceptions of digital badges in a drug information and literature evaluation course. *Currents in Pharmacy Teaching and Learning,**9*(5), 881–886. 29233319 10.1016/j.cptl.2017.05.013

[CR40] Fajri, F. A., R. K. Haribowo P, et al. (2021). Gamification in e-learning: The mitigation role in technostress. *International Journal of Evaluation and Research in Education,**10*(2), 606–614.

[CR41] García, F., Pedreira, O., et al. (2017). A framework for gamification in software engineering. *Journal of Systems and Software,**132*, 21–40.

[CR42] Gari, M. R. N., & Radermacher, A. D. (2018). Gamification in computer science education: A systematic literature review. In *ASEE annual conference and exposition, conference proceedings*.

[CR43] Garnett, T., & Button, D. (2018). The use of digital badges by undergraduate nursing students: A three-year study. *Nurse Education in Practice,**32*, 1–8. 29981502 10.1016/j.nepr.2018.06.013

[CR44] Garone, P., & Nesteriuk, S. (2019). *Gamification and learning: A comparative study of design frameworks*. Springer.

[CR45] Górska, D. (2016). E-learning in Higher Education. *The Person and the Challenges. the Journal of Theology, Education, Canon Law and Social Studies Inspired by Pope John Paul II,**6*(2), 35.

[CR46] Guérard-Poirier, N., Beniey, M., et al. (2020). An educational network for surgical education supported by gamification elements: Protocol for a randomized controlled trial. *JMIR Research Protocols,**9*(12), e21273. 33284780 10.2196/21273 PMC7744140

[CR47] Gunawan, F. E., & Jupiter,. (2018). Gamification analysis and implementation in online learning. *ICIC Express Letters,**12*(12), 1195–1204.

[CR48] Gündüz, A. Y., & Akkoyunlu, B. (2020). Effectiveness of gamification in flipped learning. *SAGE Open*, *10*(4).

[CR49] Hallifax, S., Serna, A., et al. (2019a). Factors to consider for tailored gamification. In *Proceedings of the Annual Symposium on Computer-Human Interaction in Play* (pp. 559–572). Barcelona, Spain: Association for Computing Machinery.

[CR50] Hallifax, S., Serna, A., et al. (2019b). Adaptive gamification in education: A literature review of current trends and developments. In *Lecture notes in computer science (including subseries lecture notes in artificial intelligence and lecture notes in bioinformatics)* (Vol. 11722 LNCS, pp. 294–307).

[CR51] Hervas, R., Ruiz-Carrasco, D., et al. (2017). Gamification mechanics for behavioral change: A systematic review and proposed taxonomy. In *ACM international conference proceeding series*.

[CR52] Hisham, F. B. M. N., & Sulaiman, S. (2017). Adapting gamification approach in massive open online courses to improve user engagement. *UTM Computing Proceedings Innovation in Computing Technology and Applications,**2*, 1–6.

[CR53] Huang, B., & Hew, K. F. (2018). Implementing a theory-driven gamification model in higher education flipped courses: Effects on out-of-class activity completion and quality of artifacts. *Computers and Education,**125*, 254–272.

[CR54] Hunicke, R., Leblanc, M. G., et al. (2004). MDA: A formal approach to game design and game research.

[CR55] Jianu, E. M., & Vasilateanu, A. (2017). Designing of an e-learning system using adaptivity and gamification. In *2017 IEEE international systems engineering symposium (ISSE)*.

[CR56] Júnior, E., & Farias, K. (2021). ModelGame: A quality model for gamified software modeling learning. In *15th Brazilian symposium on software components, architectures, and reuse* (pp. 100–109). Joinville, Brazil: Association for Computing Machinery.

[CR57] Kalogiannakis, M., Papadakis, S., et al. (2021). Gamification in science education. A systematic review of the literature. *Education Sciences,**11*(1), 22.

[CR58] Kamunya, S., Mirirti, E., et al. (2020). An adaptive gamification model for e-learning. In *2020 IST-Africa conference (IST-Africa)*.

[CR59] Kapp, K. M. (2012). The gamification of learning and instruction: game-based methods and strategies for training and education.

[CR60] Kapp, K. M. B. L. M. R. (2014). *The gamification of learning and instruction fieldbook: Ideas into practice*. Wiley.

[CR61] Kasinathan, V., Mustapha, A., et al. (2018). Questionify: Gamification in education. *International Journal of Integrated Engineering,**10*(6), 139–143.

[CR62] Khaleel, F. L., Ashaari, N. S., et al. (2019). An empirical study on gamification for learning programming language website. *Jurnal Teknologi,**81*(2), 151–162.

[CR63] Khaleel, F. L., Ashaari, N. S., et al. (2020). The impact of gamification on students learning engagement. *International Journal of Electrical and Computer Engineering,**10*(5), 4965–4972.

[CR64] Khalil, M., Wong, J., et al. (2018). Gamification in MOOCs: A review of the state of the art. In *IEEE global engineering education conference, EDUCON*.

[CR65] Kim, J. T., & Lee, W.-H. (2015). Dynamical model for gamification of learning (DMGL). *Multimedia Tools and Applications,**74*(19), 8483–8493.

[CR66] Kitchenham, B. (2004). *Procedures for performing systematic reviews*. Software Engineering Group Department of Computer Science, Keele University.

[CR67] Knutas, A., van Roy, R., et al. (2019). A process for designing algorithm-based personalized gamification. *Multimedia Tools and Applications,**78*(10), 13593–13612.

[CR68] Kyewski, E., & Krämer, N. C. (2018). To gamify or not to gamify? An experimental field study of the influence of badges on motivation, activity, and performance in an online learning course. *Computers and Education,**118*, 25–37.

[CR69] Landers, R. N. (2014). Developing a theory of gamified learning: Linking serious games and gamification of learning. *Simulation & Gaming,**45*(6), 752–768.

[CR70] Lavoué, E., Monterrat, B., et al. (2019). Adaptive gamification for learning environments. *IEEE Transactions on Learning Technologies,**12*(1), 16–28.

[CR71] Legaki, N. Z., & Hamari, J. (2020). Gamification in statistics education: A literature review. In *CEUR workshop proceedings*.

[CR72] Legaki, N. Z., Xi, N., et al. (2020). The effect of challenge-based gamification on learning: An experiment in the context of statistics education. *International Journal of Human Computer Studies,**144*, 102496. 32565668 10.1016/j.ijhcs.2020.102496 PMC7293851

[CR73] Llorens-Largo, F., Gallego-Durán, F. J., et al. (2016). Gamification of the learning process: Lessons learned. *IEEE Revista Iberoamericana De Tecnologias Del Aprendizaje,**11*(4), 227–234.

[CR74] Majuri, J., Koivisto, J., et al. (2018). Gamification of education and learning: A review of empirical literature. In *CEUR workshop proceedings*.

[CR75] Marín, B., Frez, J., et al. (2019). An empirical investigation on the benefits of gamification in programming courses. *ACM Transactions on Computing Education,**19*(1), 1–22.

[CR76] Mi, Q., Keung, J., et al. (2018). A gamification technique for motivating students to learn code readability in software engineering. In *Proceedings—2018 international symposium on educational technology, ISET 2018*.

[CR77] Milenković, I., Šošević, U., et al. (2019). Improving student engagement in a biometric classroom: The contribution of gamification. *Universal Access in the Information Society,**18*(3), 523–532.

[CR78] Mora, A., Riera, D., et al. (2017). Gamification: A systematic review of design frameworks. *Journal of Computing in Higher Education,**29*(3), 516–548.

[CR79] Morschheuser, B., Werder, K., et al. (2017). How to gamify? A method for designing gamification.

[CR80] Morschheuser, B., Hassan, L., et al. (2018). How to design gamification? A method for engineering gamified software. *Information and Software Technology,**95*, 219–237.

[CR81] Naik, V., & Kamat, V. V. (2015). Adaptive and gamified learning environment (AGLE). In *2015 IEEE seventh international conference on technology for education (T4E)* (pp. 7–14).

[CR82] Nicholson, S. (2012). A user-centered theoretical framework for meaningful gamification.

[CR83] Nielson, B. (2017). *Gamification mechanics vs. gamification dynamics*. Retrieved from https://www.yourtrainingedge.com/gamification-mechanics-vs-gamification-dynamics/.

[CR84] Ozdamli, S. K. A. F. (2018). A review of research on gamification approach in education.

[CR85] Pakinee, A., & Puritat, K. (2021). Designing a gamified e-learning environment for teaching undergraduate ERP course based on big five personality traits. *Education and Information Technologies,**26*(4), 4049–4067. 33613080 10.1007/s10639-021-10456-9 PMC7883948

[CR86] Park, J., De, L., et al. (2019). GAMESIT: A gamified system for information technology training. *Computers and Education,**142*, 103643.

[CR87] Pérez-López, I. J., Rivera García, E., et al. (2017). “The prophecy of the chosen ones”: An example of gamification applied to university teaching. *Revista Internacional De Medicina y Ciencias De La Actividad Fisica y Del Deporte,**17*(66), 243–260.

[CR88] Pilkington, C. (2018). A playful approach to fostering motivation in a distance education computer programming course: Behaviour change and student perceptions. *International Review of Research in Open and Distance Learning,**19*(3), 282–298.

[CR89] Rivera, E. S., & Garden, C. L. P. (2021). Gamification for student engagement: A framework. *Journal of Further and Higher Education,**45*(7), 999–1012.

[CR90] Rodríguez, I., Puig, A., et al. (2022). Towards adaptive gamification: A method using dynamic player profile and a case study. *Applied Sciences,**12*(1), 486.

[CR91] Romero-Rodriguez, L. M., Ramirez-Montoya, M. S., et al. (2019). Gamification in MOOCs: Engagement application test in energy sustainability courses. *IEEE Access,**7*, 32093–32101.

[CR92] Ropero-Padilla, C., Rodriguez-Arrastia, M., et al. (2021). A gameful blended-learning experience in nursing: A qualitative focus group study. *Nurse Education Today,**106*, 105109. 34450457 10.1016/j.nedt.2021.105109 PMC9756935

[CR93] van Roy, R., & Zaman, B. (2017). Why gamification fails in education and how to make it successful: Introducing nine gamification heuristics based on self-determination theory (pp. 485–509).

[CR94] Ryan, R., & Deci, E. (2000). Self-determination theory and the facilitation of intrinsic motivation, social development, and well-being. *The American Psychologist,**55*, 68–78. 11392867 10.1037//0003-066x.55.1.68

[CR95] Saggah, A., Atkins, A. S., et al. (2020). A review of gamification design frameworks in education. In *2020 Fourth international conference on intelligent computing in data sciences (ICDS)*.

[CR96] Saleem, A. N., Noori, N. M., et al. (2021). Gamification applications in E-learning: A literature review. *Technology, Knowledge and Learning,**27*, 139–159.

[CR97] Sanal, A. (2019). *Content gamification vs structured gamification in E-learning*. Retrieved from https://playxlpro.com/content-gamification-vs-structured-gamification-in-e-learning/.

[CR98] Sanchez, D. R., Langer, M., et al. (2020). Gamification in the classroom: Examining the impact of gamified quizzes on student learning. *Computers and Education,**144*, 103666.

[CR99] Schöbel, S., & Söllner, M. (2016). How to gamify information systems—Adapting gamification to individual user preferences.

[CR100] Schonfeld, E. (2010). SCVNGR's secret game mechanics playdeck.

[CR101] Seaborn, K., & Fels, D. I. (2015). Gamification in theory and action: A survey. *International Journal of Human-Computer Studies,**74*, 14–31.

[CR102] da Silva, R. J. R., Rodrigues, R. G., et al. (2019)."Gamification in management education: A systematic literature review. *BAR - Brazilian Administration Review*, *16*(2).

[CR103] Simões, J., Redondo, R. P. D., et al. (2013). A social gamification framework for a K-6 learning platform. *Computers in Human Behavior,**29*, 345–353.

[CR104] Smith, T. (2017). Gamified modules for an introductory statistics course and their impact on attitudes and learning. *Simulation and Gaming,**48*(6), 832–854.

[CR105] Sofiadin, A., & Azuddin, M. (2021). An initial sustainable e-learning and gamification framework for higher education. In *International conferences on mobile learning 2021 and educational technologies 2021*.

[CR106] Subhash, S., & Cudney, E. A. (2018). Gamified learning in higher education: A systematic review of the literature. *Computers in Human Behavior,**87*, 192–206.

[CR107] Swacha, J. (2021). State of research on gamification in education: A bibliometric survey. *Education Sciences,**11*, 69.

[CR108] Toda, A. M., Oliveira, W., et al. (2019). A taxonomy of game elements for gamification in educational contexts: Proposal and evaluation. In *2019 IEEE 19th international conference on advanced learning technologies (ICALT)*.

[CR109] Toda, A., Toledo Palomino, P., et al. (2020). How to gamify learning systems? An experience report using the design sprint method and a taxonomy for gamification elements in education. *Educational Technology & Society,**22*, 47–60.

[CR110] Towongpaichayont, W. (2021). A guideline of designing gamification in the classroom and its case study. *ICIC Express Letters,**15*(6), 639–647.

[CR111] Tsay, C. H. H., Kofinas, A., et al. (2018). Enhancing student learning experience with technology-mediated gamification: An empirical study. *Computers and Education,**121*, 1–17.

[CR112] Urh, M., Vukovic, G., et al. (2015). The model for introduction of gamification into E-learning in higher education. *Procedia - Social and Behavioral Sciences,**197*, 388–397.

[CR113] Uz Bilgin, C., & Gul, A. (2020). Investigating the effectiveness of gamification on group cohesion, attitude, and academic achievement in collaborative learning environments. *TechTrends,**64*(1), 124–136.

[CR114] van Gaalen, A. E. J., Brouwer, J., et al. (2021). Gamification of health professions education: A systematic review. *Advances in Health Sciences Education,**26*(2), 683–711. 33128662 10.1007/s10459-020-10000-3 PMC8041684

[CR115] van Roy, R., & Zaman, B. (2019). Unravelling the ambivalent motivational power of gamification: A basic psychological needs perspective. *International Journal of Human Computer Studies,**127*, 38–50.

[CR116] Wendy Hsin-Yuan Huang, D. S. (2013). *Gamification of education*. Rotman School of Management, University of Toronto.

[CR117] Werbach, K., & Hunter, D. (2012). For the win: How game thinking can revolutionize your business.

[CR118] Winanti, W., Abbas, B. S., et al. (2020). Gamification framework for programming course in higher education. *Journal of Games, Game Art, and Gamification,**5*(2), 54–57.

[CR119] Wongso, O., Rosmansyah, Y., et al. (2014). Gamification framework model, based on social engagement in e-learning 2.0. In *2014 2nd international conference on technology, informatics, management, engineering & environment* (pp. 10–14).

[CR120] Yamani, H. (2021). A conceptual framework for integrating gamification in elearning systems based on instructional design model. *International Journal of Emerging Technologies in Learning,**16*, 14–33.

[CR121] Yildirim, I. (2017). The effects of gamification-based teaching practices on student achievement and students’ attitudes toward lessons. *Internet and Higher Education,**33*, 86–92.

[CR122] Zainuddin, Z., Chu, S. K. W., et al. (2020). The impact of gamification on learning and instruction: A systematic review of empirical evidence. *Educational Research Review,**30*, 100326.

[CR123] Zaric, N., Lukarov, V., et al. (2020). A fundamental study for gamification design: Exploring learning tendencies’ effects. *International Journal of Serious Games,**7*(4), 3–25.

[CR124] Zhao, D., Playfoot, J., et al. (2022). An innovative multi-layer gamification framework for improved STEM learning experience. *IEEE Access,**10*, 3879–3889.

[CR125] Zichermann, G., & Cunningham, C. (2011). *Gamification by design: Implementing game mechanics in web and mobile apps*. O’Reilly Media, Inc.

